# Quantifying the carbon footprint of clinical research activities – measurement tools and methods: a scoping review protocol

**DOI:** 10.12688/hrbopenres.14092.2

**Published:** 2026-03-19

**Authors:** Dylan Keegan, Lisa Brennan, Saber Azami-Aghdash, Linda O'Neill, Peter Doran

**Affiliations:** 1 Clinical Research Centre, University College Dublin School of Medicine, Dublin, Leinster, D04YN63, Ireland; 2 Tabriz Health Services Management Research Center, Tabriz University of Medical Sciences, Tabriz, Iran

**Keywords:** carbon emission, carbon footprint, carbon measurement, clinical research, clinical trials, CO2e, narrative synthesis, scoping review

## Abstract

**Background:**

Clinical research is important for advancing medical knowledge, improving patient care, evaluating interventions, and developing new therapies. However, as with many activities, clinical research generates carbon emissions. The impact of rising emissions on the climate means that we need to examine factors that contribute to emission generation, including how these emissions are measured, as well identify mitigation strategies to reduce them. Emissions occur for various reasons, including travel for staff and research participants, the generation and disposal of research equipment, the collection and processing of laboratory samples, and data management. The aim of this review is to better understand how research activity emissions are measured, including which measurement methods and tools are identified, described and categorised..

**Methods:**

The review will include searches in seven electronic databases, including health (PubMed, Web of Science, CINAHL and Scopus) and non-health databases (EconBiz, GreenFile, and ProQuest). Primary research studies, as well as peer-reviewed grey literature sources, that may include dissertations and theses, reports, guidelines, case studies, and frameworks relevant to the aim of the review will be eligible for inclusion. Secondary research sources (i.e. reviews; systematic, scoping etc.), as well as non-peer reviewed grey literature will be excluded. Articles written entirely not in English will be excluded as the team do not have the resources to facilitate translation. Screening of titles and abstracts, and full text screening of included articles will be carried out independently by two researchers. A narrative synthesis approach will be taken to analyse the extracted data. Quality appraisal of included studies will not be carried out.

**Discussion:**

The findings will inform better understanding of methods for measurement and quantification of emissions from reported research activities. Research domains assessed and units of measurement described. These findings can benefit a range of stakeholders, particularly those working within the clinical research environment.

## Introduction

It has long been established that clinical research is important for the generation of new medical knowledge, improvement of healthcare systems, advancement of patient therapies, and evaluation and testing of new drugs and treatments. Clinical research includes various study types such as non-interventional/interventional and observational/experimental studies, that examine aspects of diseases, such as pathophysiology and response to treatment and testing. ^1^ This provides vital evidence for the safety and evaluation of treatments and therapies, advancing medical knowledge and improving our understanding of communicable and non-communicable diseases, for the improvement of patient outcomes. ^2^ Critical periods of medical research advancement, such as the COVID-19 pandemic, were vital to promptly learn about and tackle the control and prevention of diseases. ^3^ Studies of this nature, for example clinical trials, are a key cornerstone of medical research that require significant investment of time and resources. ^4^ Clinical research studies are a cornerstone of public health, and a thriving clinical research environment not only improves patient outcomes, as well as advancing medical knowledge but supports innovation and drives economic growth. ^1^

However, many activities, including healthcare and clinical research practices can generate significant carbon emissions. In the case of healthcare systems, one systematic review estimated the global impact of healthcare systems in generating carbon emissions, to be between 4–5%. ^5^ A similar systematic review compared healthcare system emissions to national emissions, reporting a mean ratio of 4.9%, with a range of 1.5% – 9.8% across the 15 included studies. ^6^ During clinical research activity, carbon emission generation can occur for various reasons, including travel for staff and research participants, the generation and disposal of research equipment, the collection and processing of laboratory samples, and storage and management of data. ^7^ In the case of clinical trials, a UK study quantified the carbon footprint of two Cancer Research UK clinical trials, the CASPS trial and the PRIMETIME trial. Trial specific activities were examined, with identification of emission factors for each activity to determine the overall carbon footprint. The CO _2_e (carbon dioxide equivalent) quantification for the CASPS trial was reported as 72 tonnes, while CO _2_e quantification for the PRIMETIME trial was reported as 89 tonnes. ^8^

These significant emission figures identified in healthcare and research environments, prompt the need for immediate action. The environmental burden of research activity globally requires assessment of these activities, particularly how to measure the emissions from these activities. We are committed to addressing the global challenge of tackling climate change and rising carbon emissions, as outlined in the Paris Climate Agreement and the United Nations Framework Convention on Climate Change (UNFCC). ^9^ It is vital that we learn how to quantify emission generation from clinical research activities, as well as learn about which activities generate emissions. In turn, this can help to identify mitigation strategies to reduce those emissions and address a key element of the global climate challenge.

### Aims and objectives

In this review we aim to investigate which methods are used to define and measure the carbon emissions from clinical research activities, as well as exploring common domains and sub-domains across these methods.

## Methods

This review will be underpinned by Arksey and O’Malley’s (2005) scoping review framework, ^10^ including elements of the Joanna Briggs Institute (JBI) scoping review guideline, ^11^ to ensure a robust, systematic approach for the conduct of scoping reviews. Arksey and O’Malley’s (2005) comprehensive framework provides a rigorous and transparent outline, which includes a five-step process for the conduct of a methodologically sound scoping review. The five-step process is as follows, identify the research question, identify relevant studies, conduct study screening, followed by data charting, and finally, perform data analysis and results reporting. The authors also recommend consideration of a sixth step, a consultation exercise, whereby relevant stakeholders may provide additional documents/literature otherwise not sourced during the search. Separately, they may provide expert insight and recommendations based on the topic area that can add value to the final reporting and recommendations. ^10^

The review protocol, where appropriate, follows the Preferred Reporting Items for Systematic review and Meta-Analysis Protocols (PRISMA-P) checklist. ^12^ Any significant protocol amendments will be updated on the HRB Open Research site.

***Research question***

For this study, the PCC (Population, Concept and Context) framework will be used, as recommended by Peters *et al.* (2021). ^11^ The PCC framework is a predominant tool in quantitative research to inform research question design, inclusion criteria and search strategies for scoping reviews. ^13^ The framework utilisation for this review is further developed below:

***P.*** Clinical research activities identified in the literature that report the generated carbon emissions.

***C.*** To explore which methods, procedures, and tools of measurement are used to quantify the carbon footprint of clinical research activities. How they are described and categorised, including comparisons between each. Carbon emission measurements (in tonnes/kg CO _2_e) and metrics associated with research activities.

***C.*** Settings where clinical research activities take place, including hospitals, clinics, research centres etc.

## Eligibility criteria

*Inclusion criteria:*

• Primary research studies.
• Peer-reviewed grey literature sources, which may include dissertations and theses, government reports, policy statements, clinical/environmental reports or guidelines.
• Case studies reporting on the measurement of carbon emissions.
• Frameworks developed that detail carbon emission measurement.
• Publications with no restricted date range.

*Exclusion criteria:*

• Secondary research sources (i.e. reviews; systematic, scoping etc.).
• Articles that are written entirely not in English as the team do not have the resources to facilitate translation.

## Search method

The search strategy for this review was informed by a specialist librarian and members of a research team in University College Dublin (UCD). The following online databases were suggested for initial inclusion: PubMed, Web of Science, CINAHL and Scopus. After further discussion with the specialist librarian, the following journal collections were also suggested to strengthen the search strategy: EconBiz, GreenFile, and ProQuest. The authors are interested in locating literature that describes the quantification of carbon footprinting for clinical research activities. As such, terms related to and including clinical research and carbon footprinting were selected. Search terms were first identified by conducting preliminary searches for these terms and identifying similar phrases, key words or other iterations of relevance. Terms unique to databases, such as MeSH and Subject Headings, were also identified and included. For full text studies included in the review, the reference lists will be searched for additional relevant studies. A descendancy approach will be taken, which is a forward literature searching method to locate articles that have cited a specific article. An ascendancy approach will also be taken, which is a backwards literature searching method that involves reviewing articles in the reference list of selected studies. ^14^ Authors of relevant articles may be contacted as needed for additional information.

This review will follow the Preferred Reporting Items for Systematic review and Meta-Analysis for Scoping Reviews (PRISMA-ScR) checklist. ^15^ A sample of the search string (including search terms, Boolean operators and fields of reference (e.g. Title/Abstract)) for the electronic database PubMed is included in Table 1.

**Table 1.** PubMed search string.

(((((((((((((“carbon footprint*”[Title/Abstract]) OR (“carbon emission*”[Title/Abstract])) OR (“co2 emission*”[Title/Abstract])) OR (“CO2e”[Title/Abstract])) OR (“CO2 equivalent”[Title/Abstract])) OR (“carbon dioxide equivalent*”[Title/Abstract])) OR (“greenhouse gas emission*”[Title/Abstract])) OR (“greenhouse gas”[Title/Abstract])) OR (“greenhouse effect*”[Title/Abstract])) OR (“environmental footprint”[Title/Abstract]))) OR (greenhouse effect [MeSH Terms])) OR (carbon footprint [MeSH Terms])) AND (((((((((((((“clinical trial*”[Title/Abstract]) OR (“interventional stud*”[Title/Abstract])) OR (“medical trial*”[Title/Abstract])) OR (“clinical stud*”[Title/Abstract])) OR (“drug trial*”[Title/Abstract])) OR (“pharmaceutical trial*”[Title/Abstract])) OR (“healthcare intervention*”[Title/Abstract])) OR (“clinical investigation*”[Title/Abstract])) OR (“randomized controlled trial*”[Title/Abstract])) OR (“non-randomized controlled trial*”[Title/Abstract])) OR (“medical research stud*”[Title/Abstract])) OR (“health research stud*”[Title/Abstract])) OR (“healthcare delivery”[Title/Abstract]))

The search will not be limited to publication dates. Only English language papers will be included as the research team do not have the resources for translatiozn.

### Screening

References from each database will be imported into Covidence, a systematic review software tool. ^16^ An institutional licence for this software was obtained through the UCD library. Duplicates will be removed prior to screening. The first reviewer (DK) and second reviewer (LB) will dual screen article titles and abstracts against the eligibility criteria using a review form to obtain a list of potential articles to include.

A list of documents will be selected and full text screening will be completed in duplicate by DK and LB. Depending on resource availability, reference list screening may be completed on some of the chosen articles, as mentioned above. Authors of the papers may be contacted for further information, if necessary. A third reviewer (LON) will be available to reconcile any differences in inclusion or exclusion decisions. The reasons for exclusion of articles included for full text screening will be provided. The search strategy and selection process for the studies will be outlined in accordance with the PRISMA-ScR statement. ^15^ This will include presentation of a PRISMA-ScR flow diagram.

### Data extraction

Using Covidence, one reviewer (DK) will use a data extraction form to extract the relevant data from the included studies. A random sample (10%) of these will be verified by a second reviewer (LB). Any conflicts that arise during the extraction process will be documented and discussed, and if resolutions cannot be decided, a third reviewer (LON) will assist.

A sample of the data and associated fields for extraction are outlined in Table 2. A pilot data extraction form will be used to extract the data, with any revisions made after consultation with the research team. A link to the form can be found in the extended data of this protocol.

**Table 2.** Study characteristics for data extraction.

Field	Characteristics
General information	• lead author • type of study/report • country of origin etc.
Details of the research activities evaluated for carbon emissions	• type of research activity
Details on the quantification of the carbon footprint associated with the clinical research activity	• name of tool • validity • domains of carbon capture • method of carbon capture etc.
Additional context	• setting • by clinical research activity e.g. clinical trial, non-trial clinical research • by domain e.g. transport, equipment, devices, data collection and storage etc.

### Risk of bias assessment

Not applicable for this review.

### Data synthesis

A narrative synthesis approach will be employed by one review member (DK) to analyse the data. ^17^ Data will be grouped in tables following the domains of the PCC framework outlined above. Study types and characteristics will be summarised together. A breakdown of clinical research activities will be outlined. Carbon emission assessment strategies, including identified tools or methods will be summarised together and examined for variances. Similarly, carbon footprint metrics and quantitative measures of environmental impact will be grouped and examined for variances.

***Analysis of subgroups or subsets***

A subgroup analysis may be performed, depending on the availability of data, which could further breakdown associated domains, examining in greater detail differences between research study types, for example clinical trials versus non-clinical trials, the carbon cost across clinical research studies, variance between research centres and non-research centres, and so on.

### Dissemination

This review will be published in an open access peer reviewed journal, as well as results being shared at relevant academic conferences and meetings. The findings from this review will be integrated into the results of a larger study that is examining the sustainability of clinical research practices.

### Protocol registration

This protocol has been registered on Open Science Framework ( https://osf.io/), an accessible open-source software repository for researchers that aids in open collaboration. The protocol can be located at the following link: OSF Registries| Quantifying the carbon footprint of clinical research activities – a scoping review protocol.

### Study status

This review is ongoing, with preliminary searches underway.

## Discussion

Clinical research is important for the generation and advancement of new medical knowledge, improving patient therapies and healthcare systems, as well as evaluation and testing of new drugs and treatments. ^1^ Research and healthcare practices can however incur negative impact on the environment through the generation of carbon emissions. ^5^ ^–^ ^7^

With the rise in emissions, it is vital to understand where they come from and how to measure them, in order to tackle the growing challenge of reducing them.

Following PRISMA-ScR guidelines, ^15^ Arksey and O’Malley’s (2005) scoping methodological framework, ^10^ and JBI’s guideline, ^11^ this review will provide a thorough summary of evidence focused on the quantification of carbon emissions related to clinical research activities. The environmental burden of research activity globally requires assessment of these activities, particularly the identification of activities that generate the most significant emissions. The review will explore which methods and metrics are being used to measure the carbon footprint of clinical research activities and explore how these factors are described and categorised.

The review will advance our understanding of quantifying carbon emissions for clinical research activities, with the results being of interest to a broad range of stakeholders, including researchers, members of the public, policy makers, funders, environmentalists and industry representatives. It may inform the development of guidelines and policies for key methods to apply when quantifying emissions and areas to target when reducing the carbon emission of research activities, a key strategic global goal.

## Ethics and consent

Ethical approval and consent were not required.

## Data Availability

No data are associated with this article.
