# Supplementary material for: Quantifying the carbon footprint of clinical research activities – measurement tools and methods: a scoping review protocol
Source: HRB Open Res. 2026 Mar 19;8:47. Originally published 2025 Apr 4. [Version 2] doi: 10.12688/hrbopenres.14092.2 (PMC13187717; doi:10.12688/hrbopenres.14092.2)
Supplement: Peer review history

# Reviewer report: Reviewer response for version 2

**DOI:** 10.21956/hrbopenres.15849.r55411

**Published:** 2026-05-19

**Authors:** Max Piffoux

**Affiliations:** 1 Hospices Civils de Lyon, Pierre-Bénite, Lyon, France

We thank the authors for launching this study. 

 I have no major comment regarding the manuscript, but I suggest to discuss (or include in the review ?) the following concept : 

 Even if the clinical research on its own has a non negligible impact, it ultimately leads to the approval of drugs (and associated care pathways) that change the standard of care for patients. These new care pathways may be much more/less emissive than the previous standard of care, and this may have a much larger carbon footprint impact than clinical research. 

 Altogether, a clinical trial may lead to the emissions of X tCO2e, but save thousand X tCO2e by changing the standard of care. 

 Although it is not directly related to the conduct of clinical research, the researchers involved into clinical research may have a strong impact on it during the design of the clinical trial, e.g. by adapting dose schedules (Immunotherapy Q3W versus Q6W, drug doses, etc). 

 Best regards

Is the study design appropriate for the research question?

Yes

Is the rationale for, and objectives of, the study clearly described?

Yes

Are sufficient details of the methods provided to allow replication by others?

Yes

Are the datasets clearly presented in a useable and accessible format?

Not applicable

Reviewer Expertise:

Medical oncology, carbon footprint of care, statistics

I confirm that I have read this submission and believe that I have an appropriate level of expertise to confirm that it is of an acceptable scientific standard.

# Reviewer report: Reviewer response for version 2

**DOI:** 10.21956/hrbopenres.15849.r54260

**Published:** 2026-04-02

**Authors:** Niamh Quann

**Affiliations:** 1 University of Leicester, Leicester, UK

This is a clear and well-written manuscript addressing an important and timely topic - the carbon footprint of clinical research activities. The methodology is clearly described and appropriate for a scoping review protocol outlining the planned approach. I look forward to seeing the results, as this is an area of personal interest and one that will be highly relevant to a broad range of stakeholders.

Is the study design appropriate for the research question?

Yes

Is the rationale for, and objectives of, the study clearly described?

Yes

Are sufficient details of the methods provided to allow replication by others?

Yes

Are the datasets clearly presented in a useable and accessible format?

Not applicable

Reviewer Expertise:

Clinical trials, research ethics, trial governance, patient and public involvement and engagement, renal.

I confirm that I have read this submission and believe that I have an appropriate level of expertise to confirm that it is of an acceptable scientific standard.
